# Face Validity and Psychometric Evaluation of the Available Oral Health-related Quality of Life Instruments: A Systematic Review

**DOI:** 10.3290/j.ohpd.a44680

**Published:** 2020-07-04

**Authors:** Shankargouda Patil, Ahmed Al Kahtani, Hosam Ali Baeshen, Abdul Wahab Alamir, Shahrukh Khan, Shilpa Bhandi, Jagadish Hosmani, A. Thirumal Raj, Amol Gadbail, Shailesh Gondivkar, Sachin Sarode, Gargi Sarode, Marco Ferrari, Kamran Habib Awan

**Affiliations:** a Associate Professor, Department of Maxillofacial Surgery and Diagnostic Sciences, Division of Oral Pathology, College of Dentistry, Jazan University, Jazan, Saudi Arabia. Study design, data search, extraction and evaluation, wrote, read and approved the final manuscript.; b Professor, Department of Restorative dental sciences, College of Dentistry, King Saud University, Riadh, Saudi Arabia. Study design, data search, extraction and evaluation, wrote, read and approved the final manuscript.; c Associate Professor, Department of Orthodontics, Faculty of Dentistry, King Abdulazziz University, Jeddah, Saudi Arabia. Study design, data search, extraction and evaluation, wrote, read and approved the final manuscript.; d Assistant Professor, Department of Maxillofacial Surgery and Diagnostic Sciences, College of Dentistry, Jazan University, Jazan, Saudi Arabia. Study design, data search, extraction and evaluation, wrote, read, and approved the final manuscript.; e Research Fellow, Centre for Rural Health, College of Health and Medicine, University of Tasmania, Australia. Data search and extraction, read and approved the final manuscript.; f Assistant Professor, Department of Restorative Dental Sciences, Division of Operative Dentistry, College of Dentistry, Jazan University, Jazan, Saudi Arabia. Data search and extraction, read and approved the final manuscript; g Assistant Professor, Oral Pathology Section, Department of Diagnostic Dental Sciences, College of Dentistry, King Khalid University, Abha, Kingdom of Saudi Arabia. Data evaluation, revised, read and approved the final manuscript manuscript.; h Lecturer, Department of Oral Pathology and Microbiology, Sri Venkateswara Dental College and Hospital, Thalambur, Chennai, India. Data evaluation, revised, read and approved the final manuscript.; i Assistant Professor, Department of Dentistry, Indira Gandhi Government Medical College and Hospital, Nagpur, Maharashtra, India. Data evaluation, revised content of the paper. read and approved the final manuscript; j Assistant Professor, Department of Oral Medicine and Radiology, Government Dental College & Hospital, Nagpur, Maharashtra, India. Data evaluation, revised manuscript content, read and approved the final manuscript.; k Professor, Department of Oral Pathology and Microbiology, Dr. D.Y. Patil Dental College and Hospital, Dr. D.Y. Patil Vidyapeeth, Pune, India. Study design, supervision, organization, and planning, read and approved the final manuscript.; l Professor, Department of Oral Pathology and Microbiology, Dr. D.Y. Patil Dental College and Hospital, Dr. D.Y. Patil Vidyapeeth, Pune, India. Study design, supervision, organization and planning, read and approved the final manuscript.; m Professor, Department of Prosthodontics & Dental Materials and Dean, School of Dental Medicine, University of Siena, Italy. Study design, supervision, organization, and planning, read and approved the final manuscript.; n Associate Professor, College of Dental Medicine, Roseman University of Health Sciences, South Jordan, Utah, USA. Study design, supervision, organization, and planning, read and approved the final manuscript.

**Keywords:** decision making, oral health, patient-based outcomes, patient safety, quality of life, questionnaire

## Abstract

**Purpose:**

A growing recognition of the importance of oral health-related quality of life (OHRQoL) has led to the development of several instruments to measure their relationship with health. The objective of this review was to update the knowledge on the general and psychometric characteristics of the instruments to measure the quality of life (QoL) related to oral health that emerged after publication in 1997 of the results of the conference ‘Measuring Oral Health and QoL’.

**Materials and Methods:**

A bibliographic search was carried out to identify publications published in January from 1998 to June 2018, using EMBASE, PubMed, Scopus, CINAHL and Web of Science databases. Specific criteria were established based on international reference frameworks for the inclusion, collection, and analysis of general and psychometric properties of the instruments.

**Results:**

233 articles were identified, of which 10 met the eligibility criteria and were included. All the instruments were multidimensional, presented psychometric properties and were mostly based on prior measurement tools and the classification of impairments and disabilities. All studies presented information on the internal consistency of their instruments. Validity to discriminate was also rated positively in all of the instruments except OHRQoL-UK instrument. Among the instruments, the criterion that was found to be least was a response to change, as only three instruments met the criteria. Reliability and construct validity criteria were also present in most of the studies.

**Conclusion:**

The dental profession has shown great progress towards a more comprehensive measurement of the oral health needs of the population, it is necessary to move from focusing on sick patients and theories of disabilities to incorporating healthy patients and resource-based theories and capacities in their measurements of OHRQoL, that would improve patient safety, quality of care and risk management, and improve clinical decision making for healthcare professionals.

Oral health is an integral component of the general health status and quality of life (QoL) of an individual.^18^ The National Oral Health Plans of various countries consider oral health a component of general health and recommend that it be part of integrated models of care for improving the oral health status and general health of people and their well-being.^8,32^ Oral conditions including caries and periodontitis are the most common chronic co-morbidities affecting the global population,^11^ the treatment of which exceeds the financial capacity and stability of the most vulnerable populations.^3,25^ These conditions have a significant impact through their associations with risk behaviours of smoking, alcohol and poor diet,^5,21^ and their contribution to the burden of chronic co-morbidities.^22,23^

The World Health Organization (WHO) highlights health as a human right.^38^ However, for governments and those responsible for health policies, it is still a low priority issue. This results in a significant number of people still experiencing inequalities in healthcare, unnecessarily, and a profound impact on their general health and in their QoL; most are widely preventable conditions and are treatable with cost-effective measures.^1^ Nikias et al^30^ reflected that we had failed to measure the impact of oral diseases on quality of people’s lives.^30^ In turn, Reisine et al^33^ and Locker et al^28^ emphasised the need for a holistic approach towards understanding the social and psychological impact of these conditions by supplementing clinical measurements of health needs with data obtained from patients in order to capture their experiences and concerns.

Based on the above considerations, a growing recognition of the importance of the QoL or patient-based outcome measures in the field of dentistry has led to the development of several instruments to measure oral health-related quality of life (OHRQoL).^2,4,35^ However, many of them are practically used only by their authors.^12^ The lack of use of QoL instruments has been argued to result from confusion and lack of understanding that exist in relation to the use of the term ‘QoL’ to evaluate the values and perceptions of patients, as well as the absence of a unified concept and an approach to their measurement.^24^

A conference paper by Frencken et al^11^ focused on health as a human right, and pointed out that this is usually a low priority for governments and those responsible for health policies. These thoughts provided important ethical groundwork and impetus for a study published in 1997, a document entitled ‘Measuring Oral Health and QoL’ which described instruments of OHRQoL.^36^ According to Locker et al,^28^ the conference document focused on presenting data on the development, evaluation, and results of the instruments, but did not address the fundamental questions: What do the instruments really measure and what are the principles on which they are based?^28^ To answer these questions, Locker et al^28^ used the criteria proposed by Gill et al^16^ to examine five of the most common instruments for measuring OHRQoL. Locker et al^28^ concluded that the claim that these instruments measure QoL is weakly justified and is in some cases inappropriate.

Knowing how and why oral health affects the QoL is useful in various ways. The development of this information could inform health professionals about the what motivates people to perform dental hygiene care, the type and pattern of use of services and programmes, as well as patient satisfaction with the treatments received.^6,7,17^ The present review arises from the apparent lack of clarity and consistency on the meaning and measurement of OHRQoL, and seeks to update the knowledge about the general characteristics and psychometrics of the instruments that emerged after the 1997 conference .^36^

## Materials and Methods

### Protocol and Registration

International Prospective Register of Systematic Reviews (PROSPERO) databases were searched for any registered protocols on a similar topic. In addition, the current systematic review was registered as a protocol with PROSPERO platform (ID: 121633). The systematic review was reported according to the Preferred Reporting Items for Systematic Reviews and Meta-Analyses (PRISMA) statement.^34^

### Focus Question

Population, Intervention, Comparison, Outcomes (PICO) criteria were employed to formulate the focus question. Participants (P) were people with oral health disorders; intervention (I) was the OHRQoL instrument; comparator (C) was the clinical assessment of the conditions; outcome (O) was the measurement of OHRQoL. The focus question was ‘What is the status of the face validity and psychometric properties of the available OHRQoL instruments?’

### Search Strategy

Detailed automated literature searches were performed in PubMed, EMBASE, Scopus, CINAHL and Web of Science using various combinations of corresponding descriptors (MeSH) and free-text terms such as ‘Oral health-related quality of life’, ‘OHRQoL instruments’, ‘oral health disorders’, ‘oral health questionnaire’. An additional search of the grey literature was carried out on Google Scholar, ProQuest, and OpenGrey. Reference lists of all included articles were manually searched to identify any potentially relevant articles. To restrict the results, the search was limited to studies published in English from January 1998 up to and including June 2018. The search strategy used for this systematic review is shown below.

### Eligibility Criteria

The following inclusion criteria were applied: (1) original peer-reviewed articles that validated the scales to measure OHRQoL or similar concepts: sociodental indicators, subjective oral health; (2) articles that present information on at least four of the following characteristics: concept to be measured, definition of the concept, domains or dimensions of the concept, information about the origin of the structure, internal consistency, test-retest reliability, validity for discrimination, validity of convergence, response to change; (3) cross-sectional, longitudinal or intervention studies.

The following exclusion criteria were applied: (1) studies that did not evaluate the OHRQoL; (2) case reports, reviews, experimental studies, short communications and personal opinions, letters to the editor, and conference abstracts.

### Study Selection and Data Extraction

Two independent reviewers (SBP; SK) screened the titles and abstracts of studies for relevant articles. Full texts of articles that fit the eligibility criteria were retrieved and reviewed by the same two reviewers. In case of disagreement, a consensus was reached through discussion. A third reviewer (KHA) was consulted in case of any disagreement.

### Data Analysis

Based on the criteria used by Gill and Feinstein,^22^ a set of 11 criteria (yes or no) was used to evaluate the face validity of all included instruments. The content details of the 11 criteria are reflected in Table 1. The psychometric properties of the included instruments were assessed using criteria laid down by Streiner and Norman,^28^ which include parameters such as internal consistency, reliability (test/retest), response to change, validity to discriminate, convergence validity and construct validity. For scoring each parameter, we used the following rating scheme: 0 (not done), − (low quality), +/− (medium quality) and + (high quality).

**Table 1:** Psychometric analysis of the included instruments

Instrument name	Internal consistency	Reliability (Test/retest)	Response to change	Validity to discriminate	Convergence validity	Construct validity	Overall score
Oral health-related quality of life instrument for dental hygiene	+	–	–	+	+	+	4/6
UK oral health-related quality of life measure (OHRQoL-UK)	+	–	–	+	–	+	3/6
Orthognathic quality of Life questionnaire (OQLQ) part I part II	+	+	+	–	+	+	5/6
Family impact of child oral and orofacial disorders (COHQOL)	+	+	–	+	+	+	5/6
Child perceptions questionnaire (CPQ11-14 COHQOL)	+	+	–	+	+	+	5/6
Parental perceptions of child oral health related quality of life (P-CPQ COHQOL)	+	+	–	+	+	+	5/6
Oral health-related quality of life index for children (CHILD-OIDP)	+	+	–	+	+	–	4/6
Parenteral perceptions of children’s oral health: Early childhood oral health impact scale (ECOHIS)	+	+	–	+	+	+	5/6
Surgical orthodontic outcome questionnaire (SOOQ)	+	+	+	+	–	–	4/6

## Results

### Study Selection

**Fig 1:**
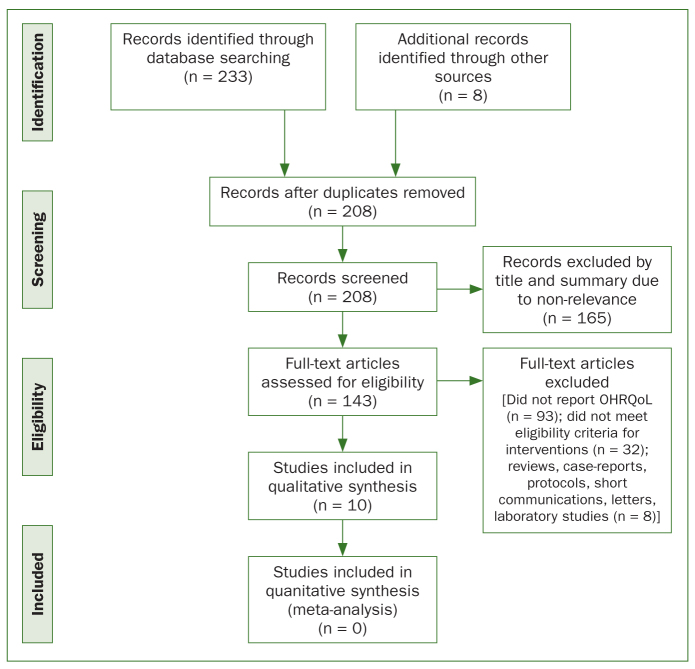
Flow diagram of literature search and selection criteria.

A total of 10 studies met the eligibility criteria and were included in the review.^9,10,13-15,20,26,27,29,31^ The subsequent review of the selected articles grouped two of the articles together to supplement the information outlined in the inclusion criteria, and since they were from the same study. The interexaminer agreement (Kappa) was 0.98 in the initial stage (title and abstract screening) and 1.00 in the following stage (full-text reading). Figure 1 presents the study selection process. Table 2 summarises the search strategy.

**Table 2:** Search strategy


Concept 1	(((((((Life Quality[MeSH Terms]) OR Health-Related Quality Of Life[MeSH Terms]) OR Health Related Quality Of Life[MeSH Terms]) OR HRQOL[MeSH Terms]) OR Oral Health Related Quality of Life) OR OHRQoL) OR OHIP*)
Concept 2	(((((questionnaire) OR question) OR item) OR tool) OR domain)
Concept 3	(((derivation) OR validation)) OR development)

### General Characteristics

All the studies included in the present analysis employed a cross-sectional study design. Regarding the characteristics of the type of instrument, four of the nine studies used generic measurement instruments which explored the health profile. Specific instruments were also found: six of the ten studies were specific in terms of the population studied,^13,14,19,20,26,31^ and five of ten studies focussed on a specific condition or health problem.^9,10,20,26,31^

All measuring instruments selected were multidimensional, and the structure of many was based on previous measurements and the classification of deficiencies and disabilities.^13,14,19,26,27,31^ Table 3 summarises the general characteristics of the nine measurement instruments analysed.

**Table 3:** Characteristics of the included instruments

Instrument name	Generic (G)* Specific (S)*	Type of population No. of participants	Mode of administration	Study type	No. of items	Dimensions or domains	Scale used	Origin of the concept
Oral health-related quality of life instrument for dental hygiene^13^	G – Health Profile S – Population	Older adult (65-95 years) Participants: 321	Self-administered	Cross-sectional	36	Status of symptoms Functional status (physical, social and psychological) Perception of oral health	Likert (5)	Health related models: OHRQ for Hygiene dental: (Wilson & Cleary HRQL model, Natural History of Disease model, Neuman’s Systems model for Nursing) and Existing measuring instrument: Oral Health Inventory profile
UK oral health-related quality of life measure (OHQoL-UK)^29^	G – Health Profile	> 18 years Participants: 390	Interview-based	Cross-sectional	16	16 key areas: eating, appearance, talking, health, comfort, encouragement, social, romance, work, finances, smile, trust, required no attention, humor, relaxation/sleep, personality.	Likert (9)	Open interviews with a population of 1865 people
Orthognathic quality of Life questionnaire (OQLQ) part I^9^ part II^10^	S – Condition or problem	Patients with dentofacial deformity (> 16 years) Participants: 88 (part I) Participants: 65 (part II)	Self-administered	Cross-sectional (part I) Longitudinal (part II)	22	Social aspect of the deformity Facial aesthetics Oral function Awareness of the facial deformity	Likert (4)	Review of the literature and in depth interviews with professionals: 10 maxillofacial, 15 orthodontists and patients
Family impact of child oral and orofacial disorders (COHQOL)^26^	S – Population and Health condition	6-14 years with oral and orofacial health problems Participants: 266 (parents-caregivers)	Self-administered	Cross-sectional	14	Family activities Parents’ emotions Family conflicts	Likert (4)	Existing OHRQoL measurement instruments: generic and specific questionnaires on health status of children that include parent-caregiver components and impact on the family of children with chronic conditions
Child perceptions questionnaire (CPQ^1,30,33,38^ COHQOL)^15^	S – Population G – Health Profile	Children (11-14 years old) Participants: 83	Self-administered	Cross-sectional	36	Oral symptoms Functional limitations Emotional well-being Social welfare	Likert (4) Likert (5)	Review of the literature (measurements of oral health and health of children) and Interviews with parents, health professionals, and children patients
Parental perceptions of child oral health related quality of life (P-CPQ COHQOL)^20^	S – Population and health condition	6-10 years and 11-14 years with oral, orthodontic and orofacial oral health problems Participants: 231 (parents-caregivers)	Self-administered	Cross-sectional	31	Oral symptoms Functional limitations Emotional wellbeing Social welfare	Likert (4) Likert (5)	Generic and specific existing instruments to measure the OHRQL of children and interviews with parents-caregivers and professionals
Oral health-related quality of life index for children (CHILD-OIDP)^14^	S – Population G – Health Profile	Children (11-12 years) Participants: 513	Interview-based	Cross-sectional	8	Impact of the disability in physical, psychological and social terms in the daily performance	Likert (3)	Existing measurement instrument: Oral impact on daily performance (OIDP) and International Classification of impairments, disabilities and handicaps (ICIDH)
Parenteral perceptions of children’s oral health: Early childhood oral health impact scale (ECOHIS)^31^	S – Population and Health condition or problem	5 years Participants: 295 (parents-caregivers)	Self-administered	Cross-sectional	13	Oral symptoms Functional limitations Emotional wellbeing Social welfare	Likert (3)	Pre-existing measuring instrument P-CPQ (focal groups and open interviews)
Surgical orthodontic outcome questionnaire (SOOQ)^27^	S – Health condition or problem.	16-58 years Participants: 95	Self-administered	Cross-sectional	33	Questions before surgery Questions after surgery Dental and facial aesthetics Social and emotional well-being	Likert (4)	Review of the literature and measuring instruments of previous OHRQoL and experts

* Generic (G): to measure the health profile. Specific (S): to measure aspects of population, disease, function, condition or problem.

### Face Validity of the Instruments

Table 4 shows the face validity results of the instruments according to the criteria established by Gill and Feinstein:^16^ ‘no’ indicated the articles that did not meet the criteria; ‘yes’, for those which complied, ‘partially’ for those which did not comply completely.

**Table 4:** Face validity of the included instruments

Instrument name	What was the objective of measurement: quality of life, health related to quality of life, other construct?	Was the meaning of the measured construct identified conceptually: quality of life, health related to the quality of life, other construct?	Were the dimensions of the measured construct identified?	Was the selection of the instrument used justified?	Were the results of multiple items, domains or instruments aggregated in a single composite index?	Were patients asked for their own overall rating for the evaluated construct: quality of life, health related to quality of life, other?	Was a distinction made between quality of life and health related to quality of life?	Were the items that comprise the questionnaire derived from qualitative interviews with those who will complete the questionnaire?	Were patients invited to supplement the list of items in the questionnaire offered by the researcher? If so, were they incorporated?	Were patients asked to indicate which items were personally important to them? If so, were they incorporated?	Did the instrument consider important events in patients’ lives?	Overall score
Oral health-related quality of life instrument for dental hygiene	Yes	No	Yes	Yes	No	Yes	No	No	No	No	No	4/11
UK oral health-related quality of life measure (OHRQoL-UK)	Yes	No	Yes	Yes	Yes	Yes	No	Yes	Partially	Partially	No	6/11
Orthognathic quality of Life questionnaire (OQLQ) part I part II	Yes	Yes	Yes	Yes	No	No	No	Yes	Yes	Yes	No	7/11
Family impact of child oral and orofacial disorders (COHQOL)	Yes	No	Yes	Yes	Yes	Yes	No	No	Partially	Partially	Yes	6/11
Child perceptions questionnaire (CPQ COHQOL)	Yes	No	Yes	Yes	Yes	Yes	No	No	Partially	Partially	Yes	6/11
Parental perceptions of child oral health related quality of life (P-CPQ COHQOL)	Yes	Yes	Yes	Yes	Yes	Yes	No	No	Partially	Partially	No	6/11
Oral health-related quality of life index for children (CHILD-OIDP)	Yes	Yes	Yes	Yes	Yes	No	No	No	No	No	Yes	6/11
Parenteral perceptions of children’s oral health: Early childhood oral health impact scale (ECOHIS)	Yes	No	Yes	Yes	Yes	Yes	No	No	Partially	Partially	Yes	6/11
Surgical orthodontic outcome questionnaire (SOOQ)	Yes	No	Yes	Yes	Yes	No	No	No	No	No	Not available	4/11

All instruments had a clear objective and dimensions of the measurements identified. In addition, all the instruments provided justification for their selection and use. Unfortunately, none of the instruments made a distinction between QoL and health-related QoL. Furthermore, most of the instruments either did not invite patients to supplement the list of items in the questionnaire or did so only partially. In terms of whether the instrument considered important events in patients’ lives, only four instruments complied.

### Psychometric Properties

Table 1 presents the results of psychometric properties according to Norman and Streiner^37^ to ensure that the instruments selected met a minimum level of psychometric properties. A plus sign (+) was placed for studies that presented information on the established criteria and a minus sign (-) for those that did not.

All the studies presented information on the internal consistency of their instruments. In addition, validity to discriminate was also rated positively in all of the instruments with the exception of the OHRQoL-UK instrument.^29^ The criterion that was found least among the instruments was a response to change, as only three instruments met the criteria.^9,10,27^ Reliability and construct validity criteria were also present in most of the studies.

## Discussion

Since the outcome of the conference ‘Measuring Oral Health and quality of life’ in 1997,^36^ at least nine instruments to measure OHRQoL have been published, which confirms the growing interest of the dental profession towards the subject.^9,10,13-15,20,26,27,29,31^ Unfortunately, however, all the instruments were published in English, thereby highlighting a lack of interest in assessing the OHRQoL in other regions of the world.

In agreement with what has been reported by Gill and Feinstein^16^ in their critical appraisal of QoL measurement, this review also showed that the development of each new instrument has become part of a complex process characterised by time-consuming, laborious steps. This includes the selection and reduction of items, pre-testing, evaluation of reproducibility and validity. From a quantitative point of view, these steps have provided the instruments with indispensable properties. However, the exploration of the qualitative properties of the studies analysed,^9,10,13-15,20,26,27,29,31^ particularly the evaluation of the face validity, did not focus on answering the question posed by Locker et al:^28^ What do the instruments measure to measure OHRQoL?

Although the present review and analysis shows a growing consensus on the multidimensionality of QoL measurement, it does not reflect explicit clarity or agreement on the terms ‘QoL’ and ‘health-related QoL’, or whether they should be taken as similar or different concepts. Likewise, most of the authors did not conceptualize the items to be measured and there was no real congruity between those who did.

It is also noteworthy that, although most of the articles showed adequate correlation between the items that constitute the dimensions of the instruments, only two of the articles derived the selection of these dimensions and the items that constitute them from qualitative interviews and analyses.^9,29^ This suggests that most of the instruments remain focused on professional opinions rather than patients, as primary users who lead the initiative and the creation of the values that govern the instruments. As long as these aspects are not addressed, the measured dimensions are likely to be inadequate to reflect the most relevant priorities, perceptions, and needs of the patients.

The instruments associated with face validity and psychometric properties are inherently subjective; hence, systematic errors could have been incorporated in the present review. The literature shows that he instruments mentioned in the present study are widely used, and to the best of our knowledge, the literature does not contain completely objective instruments. However, the review was carried out under specific, clearly established and confirmed criteria before inception. This allowed minimising biases regarding evaluations of quantitative and qualitative psychometric properties of instruments to measure OHRQoL.

In general, the problems of confusion and lack of consensus on the term OHRQoL require that a debate be conducted on how to obtain greater clarity and encompass the most appropriate domains in the instrument.

## Conclusions

While the dental profession has made great progress towards more comprehensive measurement of the oral health needs of the population, it is necessary to move from focusing on sick patients and theories of disabilities to incorporating healthy patients and resource-based theories and capacities in measurements of OHRQoL. Similarly, the challenge of expanding the use of instruments to measure OHRQoL persists. Although some instruments initial present adequate psychometric properties, they require validation through use in diverse populations and contexts. Finally, there is an urgent need to conduct research on the subject in other countries to identify and/or modify scales to adapt them to the given context and characteristics of the specific healthcare system, as well as the socioeconomic and cultural aspects of a given population.
